# Pulmonary hypertension: Spectrum of disease, clinical presentation and treatment outcomes at the main respiratory pulmonary hypertension clinic in KwaZulu-Natal Province, South Africa

**DOI:** 10.7196/AJTCCM.2021.v27i1.118

**Published:** 2021-03-09

**Authors:** M Dahim, M Mitha, C Connolly, K Nyamande

**Affiliations:** 1 Department of Pulmonology and Critical Care, Groote Schuur Hospital, Cape Town, South Africa; 2 Department of Pulmonology and Critical Care, Inkosi Albert Luthuli Central Hospital, Durban, South Africa; 3 Department of Biostatistics, School of Nursing and Public Health, University of KwaZulu-Natal, Durban, South Africa

**Keywords:** pulmonary hypertension, HIV, KwaZulu Natal

## Abstract

**Background:**

There are many causes of pulmonary hypertension (PH). However, the aetiology, management and treatment outcomes in South Africa (SA), which has a high burden of HIV, are lacking in the literature.

**Objectives:**

To characterise patient demographics, aetiology, clinical presentation and management of patients presenting to the only government-funded PH clinic in Durban, SA.

**Methods:**

We retrospectively reviewed electronic charts of patients with confirmed PH who attended the respiratory PH clinic between 2011 and 2018. Demographic and clinical data, symptoms, pulmonary function testing, pulmonary artery pressure on echocardiography and treatment were analysed. Patients with group 2 PH were excluded from the present study as they were managed by cardiologists.

**Results:**

We identified 93 patients with confirmed PH and the majority were female (82.8%; n=77). The majority of the patients were between the ages of 30 and 39 years at the time of diagnosis. Most patients were black African (64.5%; n=60), followed by Indians (26.9%; n=25) and whites (8.6%; n=8). The most common cause of PH was group 1 (75%; n=70), followed by group 4 (13%; n=12) and then group 3 (12%; n=11). HIV-associated PH accounted for 27% of all patients and was the main cause of PH in those classified in group 1 (38%; n=29). Two-thirds (66%) of patients were treated with sildenafil, the only treatment that was available. Patients on treatment showed significant improvement indicated by the World Health Organization functional class, mean 6-minute walk test and reduction in mean pulmonary artery pressure on echocardiography

**Conclusion:**

HIV-associated PH is the most common cause of PH in SA. Sildenafil, the only drug available in our setting, is beneficial to most patients with PH.

## Background

Pulmonary arterial hypertension (PAH) is an uncommon and lifethreatening disease with limited literature regarding its prevalence and aetiology in South Africa (SA).^[1,2]^ Pulmonary hypertension (PH) was initially classified as a mean pulmonary artery pressure (mPAP) ≥25 mmHg at rest; however, this was changed at the 6th World Symposium on Pulmonary Hypertension in 2018 to be mPAP >20 mmHg at rest.^[3]^ It is broadly classified into 5 groups by the World Health Organization (WHO) based on similar clinical and pathophysiological characteristics.^[4]^

There is paucity of literature regarding the epidemiology of PH in sub-Saharan Africa. A recent study by Davies van Es *et al*.^[2]^ analysed the PH registry at Groote Schuur Hospital in Cape Town. The Pan African Pulmonary Hypertension Cohort (PAPUCO) is the largest registry in Africa, comprising four countries and 209 adults in total to analyse PH in Africa.^[5]^

To the best of our knowledge, the present study is the first to evaluate the causes and outcomes of PH in adults in KwaZulu-Natal (KZN) Province, SA.

## Methods

We undertook a retrospective electronic chart review of patients attending the respiratory PH clinic at Inkosi Albert Luthuli Central Hospital (IALCH) between January 2011 and December 2018. IALCH is the only public-sector hospital treating patients with PH in the KZN Province.

Patients who were 12 years and older were included in the study. Patients who had group 2 PH were excluded, as they were managed by the cardiologists and not in the respiratory PH clinic.

Demographic data included age, sex and race. Clinical data comprised the aetiology of PH as classified by the European Society of Cardiology (ESC)/European Respiratory Society (ERS). Data collected included presenting symptoms, WHO functional class pre- and post-treatment, specific treatment for PH, comorbidities and HIV status.

Special investigations included high-resolution computed tomography (HRCT), pulmonary function test (PFT), pre- and post-treatment 6-minute walk test (6MWT) and echocardiography

Ethical approval for the study was granted by the University of KwaZulu-Natal Biomedical Research Ethics Committee (ref. no. BE578/18). All participants’ details were kept confidential and their clinical details identified and collated on a datasheet.

### Statistical analysis

The percent change in forced vital capacity (FVC), forced expiratory volume in 1 second (FEV_1_) between the initial reading and the last reading was calculated and then categorised into deteriorated (<–10%), static (–10% - +10%) and improved (>10%). Dyspnoea was categorised as any improvement in dyspnoea grade before and at any point after treatment. The mean difference between pre- and post-6MWT and pulmonary artery systolic pressure (PASP) was compared using a paired t-test. Fisher’s exact test was used to compare categorical variables between treated v. untreated patients. Statistical significance was regarded as a p<0.05 unless otherwise stated. Stata software version 15 (StataCorp., USA) was used for statistical analysis.

## Results

A total of 93 patients were included in the study, with a female preponderance (82.8%; n=77). Most patients were aged 30 - 39 years, followed by those who were 50 - 59 years.

**Fig. 1:**
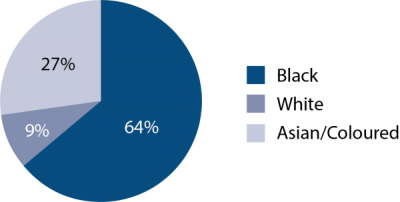
Racial demographics of patients with pulmonary hypertension.

Almost two-thirds of patients were black African (64.5%; n=60), followed by Indians (26.9%; n=25) and whites (8.6%; n=8) (Fig. 1).

**Fig. 2:**
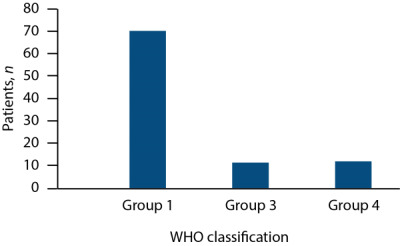
World Health Organization classification distribution of pulmonary hypertension.

Three-quarters (75%; n=70) of patients were classified in group 1, 2% (n=11) in group 3 and 13% (n=12) in group 4 according to the WHO classification (Fig. 2). Patients that belonged to group 2 were excluded from this study. Patients in group 1 were classified based on history, laboratory and radiological investigations. Patients in group 3 were classified after review of their clinical history with HRCT scan features suggesting lung disease as well as pulmonary function tests. Patients in group 4 were also classified after an extensive review of their clinical history, CT pulmonary angiography in some patients, and some patients (n=3) also had ventilation-perfusion scans taken (Fig. 2).

Group 1 patients comprised the following: 38% (n=29) had PH secondary to HIV; 33% (n=23) had idiopathic PAH; and 24% (n=18) had PH secondary to connective tissue disease.

The majority of patients with PH secondary to HIV were on antiretroviral treatment. Viral load was recorded in 26 patients and it was suppressed (<50 copies/mL) in about half of these patients (47%; n=15). The median (interquartile range (IQR)) CD4 count in HIV-positive patients (IQR)) of 461 (14 - 1 558) cells/µL.

In our study population, the most common cause of PH secondary to connective tissue disease was systemic lupus erythematosus (SLE) occurring in 52% (n=10) of patients and systemic sclerosis (SS) in 21% (n=4) of patients. There were two patients with mixed connective tissue disease (MCTD) and undifferentiated connective tissue disease (UCTD), respectively

### Presenting symptoms

**Fig. 3:**
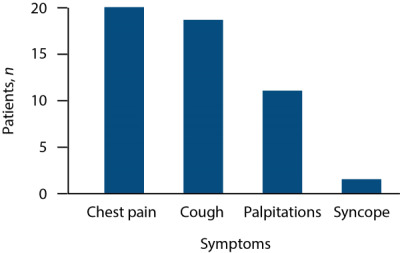
Symptom presentation of patients with pulmonary hypertension.

Exertional dyspnoea was the main symptom at presentation for all patients. Other symptoms included chest pain (21%), cough (20%), palpitations (12%) and syncope (1%) (Fig. 3). WHO functional class was documented at presentation with majority being class II (48%) and III (47%).

### High-resolution CT scan (HRCT)

The majority of patients (72%; n=66) had normal scans, 8% (n=7) had nonspecific interstitial pneumonia (NSIP), 5% (n=4) had usual interstitial pneumonia (UIP), 11% (n=10) had chronic thromboembolic pulmonary hypertension (CTEPH) and 5% (n=5) had other findings. In the NSIP subgroup, five patients were diagnosed with SLE, one with MCTD and one with UCTD. In the UIP subgroup, two patients were diagnosed with SS, one with SLE and the other with MCTD.

### Pulmonary artery systolic pressure on echocardiography

PASP was recorded in all patients via echocardiogram at the time of referral, with a median (IQR) reading of 63 (28 - 110) mmHg. Patients with a tricuspid regurgitant jet >2.8 m/s or right ventricular systolic pressure above 30 mmHg were indicative of PH. Right heart catheterisations were not done as the service was not offered routinely even though it is the gold standard in determining pulmonary pressures.

### Pulmonary function testing

PFT was documented in 86% (n=80) of the patients. The majority of patients 81% (n=65) had a restrictive pattern, 17.5% (n=14) had normal PFT results and only one case had an obstructive pattern. A total of 83 patients underwent an initial 6MWT and the mean (standard deviation (SD)) distance was 357 (12) m.

### Treatment outcomes

A total of 61 patients received specific treatment for PH. Almost all the patients (n=60) were on sildenafil, a phosphodiesterase-5 inhibitor, at a dose of 20 mg three times daily, and one patient was on a calcium channel blocker (amlodipine).

Of the 61 cases on treatment, 55 were from group 1 and six from group 4. The patient’s symptoms, FVC, FEV_1_, 6MWT, PASP were analysed prior to starting treatment and after commencing treatment at any point between 3 months and 2 years.

### Change in functional class

There was a statistically significant (p=0.04) improvement in at least one grade of dyspnoea in more than two-thirds (68%; n=44) of patients.

### Pulmonary artery systolic pressure

**Fig. 4:**
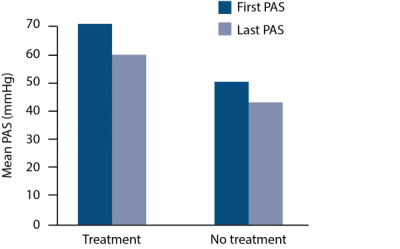
Change in mean pulmonary artery systolic pressure (PASP) preand post-treatment (p=0.006).

Among the 55 patients who had a PASP pre- and post-treatment, there was a significant decrease in the mean PASP of 11 mmHg. There was a statistically significant difference (p=0.006) between the mean (SD) pre- and post-treatment PASP of 69 (20) mmHg and 58 (21) mmHg, respectively (Fig. 4).

### 6-minute walk test

**Fig. 5:**
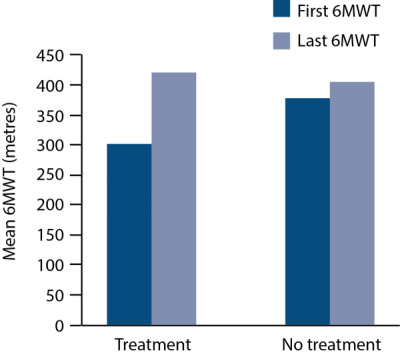
Mean 6-minute walk test (6MWT) pre- and post-treatment (p<0.001).

The majority of the patients (77%; n=47/61) on treatment had pre- and post-treatment values for 6MWT. The initial mean (SD) 6MWT was 291 (130) m and the mean (SD) post-treatment was 408 (128) m. The mean difference of 116 m was statistically significant (p<0.001) (Fig. 5).

## Discussion

The present study aimed to highlight the causes of PH in KZN Province and this is the second study to the best of our knowledge to describe PH in SA. The majority of the patients were in the 30 - 39-year age group. Most of the patients were black African, followed by Indians and then whites. The majority of patients were female, which is consistent with international trends^[5]^ and the findings of the Groote Schuur registry study.^[2]^

The racial balance did not follow provincial demographics, with ethnic Indians having the second highest prevalence although they are the minority in the province. This may suggest a genetic predisposition to the disease or a possible referral bias.

The majority of the patients were group 1, with the remainder equally shared between groups 3 and 4. This is similar to the findings of the Groote Schuur registry. However, this differs from the findings of PAPUCO study,^[5]^ where the majority of patients were in group 2. These patients were excluded from this study as they are managed by cardiologists.^[2,5]^ We analysed non-cardiac PH. HIV-associated PH was more prevalent than idiopathic PH. HIV-associated PH constituted ~19% of the study population and 38% of patients in group 1. The present study highlights that HIV is probably the most common cause of disease in KZN Province and SA, as found in the Groote Schuur registry.^[2]^ However, it contrasts with studies from Europe,^[7,8]^ where the prevalence of HIV-associated PH is ~0.5%. There have not been many studies done in high-HIV prevalent areas to determine the true prevalence of HIV-associated PH. Literature from the USA differs from Europe, with the prevalence being <10% in Europe and 35% in the USA.^[9]^ Due to the retrospective nature of this study, and the temporal relationship between HIV and the development of PH, we could not establish a relationship between PH, CD4 count and viral load.

The most common cause of PAH due to connective tissue disease was SLE rather than SS. This is in contrast with the findings from the REVEAL study.^[10]^ However, studies in some Asian countries^[11]^ show that SLE is more prevalent than SS in PAH. There have been no studies to determine if SS or SLE is more prevalent in the SA population. It is possible that SLE is more prevalent, hence accounting for the higher SLE-associated PAH.

Medical treatment for PH is directed towards patients with PAH, as well as CTEPH patients, who are not eligible for pulmonary endarterectomy. Our patients only received single drug therapy with sildenafil as it is the only available treatment, and these patients showed improvement in functional class, 6MWT distance as well as a reduction in PASP. This is consistent with other studies in which sildenafil was used.^[12–14]^ The use of combination therapy has been recommended by the ESC/ERS guidelines as multiple pathways are targeted and this has an impact on survival.^[15]^ However, in the public sector in KZN, there is no other drug available besides sildenafil and its use has been shown to be beneficial to the patients, highlighting its effective role as initial monotherapy.

Right-heart catheterisation is recommended in the assessment and management of these patients according to ESC/ERS guidelines. We were unable to perform right heart catheterisation to fully assess these patients with regards to precise pressures and vasoreactivity testing .

### Study limitations

The present study is an electronic retrospective chart review with missing data, and this may have compromised the results. We conducted the study at the main respiratory hypertension clinic, which could have resulted in referral bias. We could have missed more patients due to the recent change in the diagnostic criteria.

## Conclusion

This is the first study to the best of our knowledge to describe the demographic distribution and treatment outcomes of patients with PH in SA, specifically in the KZN Province. The present study revealed that HIV is the greatest contributor to PH in the province, followed by idiopathic PAH. SLE is the most common contributor in patients with connective tissue disease-associated PH. There is a need for right heart catheterisation for optimal diagnosis and management of patients in our setting. The establishment of multidisciplinary teams comprising cardiologists, pulmonologists and radiologists will be extremely beneficial in this endeavour. There is also a greater need for engagement with the provincial health department and the private sector to obtain more medications to allow for add-on and combination therapy in KZN. Further studies are required in KZN, and the rest of SA, to assess optimal clinical management of PH. Finally, large-scale studies are also required in sub-Saharan Africa because of the high prevalence of HIV infection.
